# Reviewers

**DOI:** 10.1111/jdi.13414

**Published:** 2020-11-04

Felix Aberer

Norio Abiru

Samrein B. M. Ahmed

Ji‐Hyun Ahn

Vasudha Ahuja

Jun Aida

Toru Aizawa

Gulali Aktas

Aydin Akyuz

Sirajudeen S. Alavudeen

Rachael Allen

Noe Alvarado

Takatoshi Anno

Keizo Anzai

Shigeru Aoki

Hidenori Arai

Keiko Arai

Atsushi Araki

Shin‐ichi Araki

Naoko Arata

Hiroshi Arima

Osamu Arisaka

Noriko Asahara

Shunichiro Asahara

Nick Ashton

Yoshimasa Aso

Vasilios Athyros

Takuya Awata

Motoharu Awazawa

Masayuki Baba

Ji‐Cheol Bae

Yuqian Bao

Beatriz Barquiel

Nathan Berger

John Blaikley

Aldo Bonaventura

Alexandro Bonifaz

Ryotaro Bouchi

Christina Breidenassel

Steven Brenner

Soren Brunak

Magnus Bruze

Xiaoling Cai

Soner Cander

Sven Magnus Carlsen

Luke Carroll

Ali Riza Cenk Celebi

Hyun Wook Chae

Ali J. Chakera

Christine L. Chan

Juliana Chan

Te‐Fu Chan

Emily Chang

Jer‐Ming Chang

Yi‐cheng Chang

Sanjay Chatterjee

Cheng‐Hsu Chen

Harn‐Shen Chen

Judy Chen

Min Chen

Qingjie Chen

Szu‐Tah Chen

Wei Chen

Ching Lung Cheung

Pik To Cheung

Boon‐How Chew

Siew Mooi Ching

Ken Chiu

Kae Won Cho

Kyu Yong Cho

Sung Hee Choi

Won‐Il Choi

Wing Sun Chow

Mark P. Christiansen

Nain‐Feng Chu

Tsung‐Ju Chuang

Daisuke Chujo

Ho Yeon Chung

Dana Ciobanu

Massimo Cirillo

Rachel Climie

Makoto Daimon

Undurti Das

Buyzere Marc De

Asma Deeb

Takahisa Deguchi

Marloes Nitert Dekker

Rick Dobrowsky

Kentaro Doi

Gracia‐Ramos Abraham Edgar

Nathan Efron

Kazuo Eguchi

Nabil Eid

Gerges Najwa El

Nabil El‐Naggar

Masanori Emoto

Johan Eriksson

Fabio Facchinetti

Gabor Ferneisz

Paul Fernyhough

Ele Ferrannini

Jose L. Flores‐Guerrero

Luisa Frizziero

Chia‐Po Fu

Kazuya Fujihara

Toshihito Fujii

Rumi Fujikawa

Junji Fujikura

Kei Fujimoto

Kosuke Fujimoto

Shimpei Fujimoto

Shiho Fujisaka

Tomomi Fujisawa

Yoshihito Fujita

Yukihiro Fujita

Yoshio fujitani

Yukio Fujiwara

Kei fukami

Maki Fukami

Astunori Fukuhara

Michiaki Fukui

Tomoyasu Fukui

Shingo Fukuma

Nobuaki Funahashi

Masato Furuhashi

Shinya Furukawa

Hiroto Furuta

Xin Gao

Isabel Garcia‐Garcia

Abed Ghavami

Pramit Ghosh

Reza Ghotaslou

Pieter Gillard

Tomohito Gohda

Atsushi Goto

Haotian Gu

Yashdeep Gupta

Kyoung Hwa Ha

Yusuke Hada

Samy Hadjadj

Yoji Hamada

Kazuyuki Hamaguchi

Masahide Hamaguchi

Hidetaka Hamasaki

Jin Han

Seung Jin Han

Ying Han

Kazuo Hara

Mariko Harada‐Shiba

Ai Harashima

Shin‐Ichi Harashima

Hans‐Ulrich Häring

Noriyasu Hashida

Koshi Hashimoto

Mohamed Azmi Hassali

Mohamed Hassanein

Jun Hata

Yuji Hataya

Akinori Hayashi

Yoshitaka Hayashi

Yasuaki Hayashino

Mudiyanselage Meththananda Herath

Tatsuhito Himeno

Hiroki Hirai

Tsutomu Hirano

Takumi Hirata

Jun Hirayama

Takahisa Hirose

Inoue Hiroshi

Yushi Hirota

Mikano Hishiki

Jens Juul Holst

Yukio Horikawa

Masayuki Hosoi

Kazuyoshi Hosomichi

Tetsuya Hosooka

Chang‐Hsun Hsieh

Cheng Hu

Gang Hu

Huanhuan Hu

Xinqun Hu

Chia‐Luen Huang

Jee‐Fu Huang

Kuo‐Chin Huang

Weidong Huang

Elaine Hui

Xiaoyan Hui

Tai‐Ho Hung

Humaira Hussein

You‐Cheol Hwang

Chii‐Min Hwu

Jon Hyett

Yasuo Ido

Matias Iglicki

Katsumi Iizuka

Takayuki Ikeda

Hiroshi Ikegami

Satoru Ikenoue

Junta Imai

Fumiaki Imamura

Minako Imamura

Toyoshi Inoguchi

Daisuke Inoue

Kazuo Inoue

Junichiro Irie

Shun Ishibashi

Yasushi Ishigaki

Hisamitsu Ishihara

Yuichi Ishihara

Hiroshi Ishii

Mohammad Safiqul Islam

Tatsuya Iso

Masataka Ito

Yoshiya Ito

Hiroshi Itoh

Masato Iwabu

Toshio Iwakura

Naoko Iwasaki

Masanori Iwase

Takanori Iwata

Hak Chul Jang

Edward Janus

Mu'taman Jarrar

Ja Young Jeon

Jae‐Han Jeon

Sang‐Man Jin

Chang Hee Jung

Tetsuya Kakuma

Hiromitsu Kanamori

Naotetsu Kanamoto

Keizo Kanasaki

Akio Kanazawa

Ippei Kanazawa

Naomi Kane

Shizuka Kaneko

Hideaki Kaneto

Hee Taik Kang

Hideki Katagiri

Sayaka Katagiri

Naoto Katakami

Koichi Kato

Yoshiro Kato

Tomoko Kawai

Toshihide Kawai

Dan Kawamori

Tomoyuki Kawamura

Daiji Kawanami

Eiji Kawasaki

Jyunji Kawashima

Mykola Khalangot

Yoshiaki Kido

Takeshi Kikuchi

Toru Kikuchi

Bu Kyung Kim

Chong Hwa Kim

Chul‐Hee Kim

Eun Ky Kim

Hail Kim

Jae Hyeon Kim

Jaetaek Kim

Kyoung Min Kim

Kyung‐Soo Kim

Mee Kyoung Kim

Nan Hee Kim

Sang Soo Kim

Sang Yong Kim

Munehiro Kitada

Tadahiro Kitamura

Arihiro Kiyosue

Atsushi Kiyota

Tetsuro Kobayashi

Mitsuhisa Komatsu

Tatsuya Kondo

Bokyung Koo

Masaya Koshizaka

Daisuke Koya

Hidenori Koyama

Peter Kristensen

Andrzej Krolewski

Hiroyuki Kubota

Naoto Kubota

Shinji Kume

Shoen Kume

Makoto Kurano

Akio Kuroda

Akira Kuroe

Takeshi Kurose

Toru Kusakabe

Hitoshi Kuwata

Masafumi Kuzuya

Sin Man Lam

Ting‐I Lee

Da Young Lee

Dae Ho Lee

Eun Young Lee

I‐Te Lee

Mei‐Yueh Lee

Meng‐Chih Lee

Nami Lee

Paul Lee

Seung Hwan Lee

Shaun Lee

Ting‐Wei Lee

Won‐Young Lee

Yau‐Jiunn Lee

Yongho Lee

Yù Huì Lee

Shaoqing Lei

Qian LI

Xia Li

Yuxiu Li

Xinxue Liao

Chia‐Huei Lin

Chia‐Hung Lin

Hugo You‐Hsien Lin

Kun‐Der Lin

Liang‐Yu Lin

Mao‐Shin Lin

Ming Lin

Shih‐Yi Lin

Shin‐Jong Lin

Shin‐Yu Lin

Randie Little

Feng‐Hsuan Liu

Ming Liu

Wanli Liu

Anna Long

Peirong Lu

Jianhua Ma

Audrey Macdonald

Norikazu Maeda

Hiroshi Maegawa

Tanmay Mahapatra

Loey Mak

Noboru Makabe

Shinya Makino

Jana Makuc

Rachid Malek

Rayaz Malik

Boris Mankovsky

Gomez Ana Maria

Maurizio Marra

Hiroaki Masuzaki

Ikuro Matsuba

Masafumi Matsuda

Munehide Matsuhisa

Munetaka Matsuhisa

Michihiro Matsumoto

Takeshi Matsumura

Taka‐aki Matsuoka

Kishor Mazumder

Shu Meguro

Ruilope Luis Miguel

Takashi Miki

Takayuki Miki

Selvaraj Miltonprabu

Isao Minami

Takashi Minami

María Miranda

Hirofumi Misu

Tomoya Mita

Junnosuke Miura

Teruki Miyake

Kenichi Miyako

Takeshi MIyatsuka

Hideaki Miyoshi

Hiroki Mizukami

Piotr Mlynarz

Mie Mochizuki

Mouhand Mohamed

Jun Sung Moon

Sun Joon Moon

Yousef Moradi

Thyago Proenca de Moraes

Andrey Morgun

Yasumichi Mori

Jun Morinaga

Katsutaro Morino

Tomoaki Morioka

Yoshiaki Morishita

Wali Muhammad

Seiichi Munesue

Chieko Murakami

Tomoaki Murakami

Tsukasa Murakami

Takashi Murata

Alyson Myers

Seiho Nagafuchi

Yoshio Nagai

Mototsugu Nagao

Shoichiro Nagasaka

Terumasa Nagase

Masao Nagata

Tomohisa Nagata

Takeshi Naitoh

Tomoko Nakagami

Kei Nakajima

Toshiaki Nakajima

Akinobu Nakamura

Mieko Nakamura

Nobuhisa Nakamura

Shuhei Nakanishi

Eitaro Nakashima

Koshi Narasaki

Koji Naruishi

Mitsuhide Naruse

Masami Nemoto

Jack Ng

Monika Niewczas

Toshiharu Ninomiya

Masahiro Nishi

Kenro Nishida

Nao Nishida

Tetsuo Nishikawa

Rimei Nishimura

Wataru Nishimura

Yoshihiko Nishio

Shinta Nishioka

Mitsuhiko Noda

Hiroyuki Nodera

Takashi Nomiyama

Takuo Nomura

Sedigheh Nouhjah

Makiko Ogata

Sumito Ogawa

Yoshihiro Ogawa

Masahito Ogura

Ken Ohashi

Haruya Ohno

Masahiro Ohsawa

Mitsuru Ohsugi

Masayuki Ohta

Yasuharu Ohta

Sumio Ohtsuki

Yoichi Oikawa

Yosuke Okada

Tsuyoshi Okura

Yukiko Onishi

Hiroshi Onuma

Haruhiko Osawa

Takashi Oshiro

Tsuguhito Ota

Horng‐Yih Ou

Motoshi Ouchi

Nobuaki Ozaki

Jiemin Pan

Kyong Soo Park

Se‐Jun Park

Milan Perovic

Giorgina Piccoli

May Ee Png

Sofie Prikken

Flavia Prodam

Pirkko J Pussinen

Hanbo Qiu

Hua Qu

Ajenthen Ranjan

Gerry Rayman

Cme Reynolds

Lene Ringholm

Anitha Risberg

Lauren Rodgers

Celia Rodrigues

Ishibashi Ryoichi

Paola Sacerdote

Yoshifumi Saisho

Kazuhiko Sakaguchi

Juro Sakai

Masaya Sakamoto

Hiroshi Sakaue

Ichiro Sakuma

Kenichi Sakurai

Masaru Sakurai

Clara Bik San Lau

Sheikh Mohd Saleem

Jose Luis Sanchez‐Quesada

Sang‐Man Sang‐Man

Kazunori Sango

Motoaki Sano

Juliann Saquib

Hideyuki Sasaki

Takayoshi Sasako

Hiroaki Satoh

Jo Satoh

Maryam Sattari

Yosuke Seki

Dinesh Selvarajah

Vichai Senthong

Jamil Shah

Viral Shah

Neel Sharma

Jonathan E. Shaw

Yi‐Jing Sheen

Wayne Sheu

Shyang‐Rong Shih

Kenichi Shikata

Michio Shimabukuro

Akira Shimada

Yoshihiro Shimazaki

Ikki Shimizu

Masashi Shimoda

Masayuki Shimoda

Iichiro Shimomura

Jae Yong Shin

Jun Shirakawa

Channabasappa Shivaprasad

Nobuhiro Shojima

Xiang Shu

Angelos K. Sikalidis

Jan Skupien

Carlo Eduado Medina Solis

Masakatsu Sone

Ki‐Ho Song

Su‐Jeong Song

Xue Song

Renato Teixeira Souza

Vincenza Spallone

Ian Spence

Julie Stoy

Jian‐Bin Su

Sheng‐Chiang Su

Yu‐Wen Su

Shigetaka Sugihara

Ken Sugimoto

Masahiko Sugimoto

Takashi Sugiyama

Takehiro Sugiyama

Cheuk‐Kwan Sun

Mingzhong Sun

Zi Sun

Zilin Sun

Atushi Suzuki

Ken Suzuki

Ryo Suzuki

Shigeru Suzuki

Yasuharu Tabara

Yuji Tajiri

Mitsuyoshi Takahara

Kazuma Takahashi

Toshinari Takamura

Shin Takasawa

Yasunori Takata

Takeshi Takayanagi

Fumihiko Takeuchi

Wing Hung Tam

Yoshikazu Tamori

Kouichi Tamura

Yoshifumi Tamura

Bee Kang Tan

Kok Hian Tan

Katsuya Tanabe

Katsuyuki Tanabe

Daisuke Tanaka

Nagaaki Tanaka

Nobue Tanaka

Jun Tao

Shimosawa Tatsuo

Shuji Terai

Yasuo Terauchi

Jiaobing Tian

Kazuyuki Tobe

Atsuhito Tone

Keiichi Torimoto

Masao Toyoda

Dimitrios Tsilingiris

Satoru Tsujii

Shin Tsunekawa

Yuya Tsurutani

Wen‐Jun Tu

Yu‐Kang Tu

Jaakko Tuomilehto

Yasuko Uchigata

Kohjiro Ueki

Hiroaki Ueno

Satoshi Ugi

Hiroyuki Umegaki

Akira Umemura

Hiroyuki Unoki‐Kubota

Tatsuhiko Urakami

Isao Usui

Ryota Usui

Kazunori Utsunomiya

Roxana Valdes‐Ramos

Istvan Vassanyi

Kavita Venkataraman

Nathalie Viguerie

Francesc Villarroya

Jun Wada

Chaofan Wang

Chih Yuan Wang

Jann‐Tay Wang

Jianxiong Wang

Jun‐Sing Wang

Miao Wang

Xiao Qun Wang

Yeli Wang

Scott White

Paweł Wiech

Mark Willems

Jong Chul Won

Kyu Chang Won

Connie Woo

Bin Wu

Chung‐Ze Wu

Ling Wu

Min Xia

Jian Xiao

Jianzhong Xiao

Xinhua Xiao

Yanqiu Xing

Lingling Xu

Wen Xu

Xiaolei Xu

Yong Xu

Zhangrong Xu

Chihiro Yabe

Kunimasa Yagi

Hiroaki Yagyu

Tetsuya Yamada

Yuichiro Yamada

Sho‐ichi Yamagishi

Kanji Yamaguchi

Masahiro Yamamoto

Takeshi Yamamoto

Yasuhiko Yamamoto

Ritsuko Yamamoto‐Honda

Shunsuke Yamane

Tomoyuki Yamasaki

Satoshi Yamashita

Tomoya Yamashita

Ichiro Yamauchi

Yuji Yamazaki

Fuhua Yan

Jinhua Yan

Yuan‐Horng Yan

Aimin Yang

Fei Yang

Gangyi Yang

Jichun Yang

Tse‐Yen Yang

Xilin Yang

Zu‐yao Yang

Hisafumi Yasuda

Hitoshi Yasuda

Yutaka Yatomi

Hiroshi Yatsuya

Ester Yeoh

Shiu Lun Au Yeung

Jun Yin

Norihide Yokoi

Hiroshi Yokomichi

Hideto Yonekura

Tohru Yorifuji

Yoshihiro Yoshimura

Narihito Yoshioka

Dingyun You

Liping Yu

Yanggang Yuan

Agnieszka Zawada

Dongyu Zhang

Guangxiang Zhang

Qiu Zhang

Sen Zhang

Yang Zhang

Kangren Zhao

Xiaolong Zhao

Ying‐Yong Zhao

Hongting Zheng

Jian Zhou

Naiming Zhou

Zhiming Zhu

